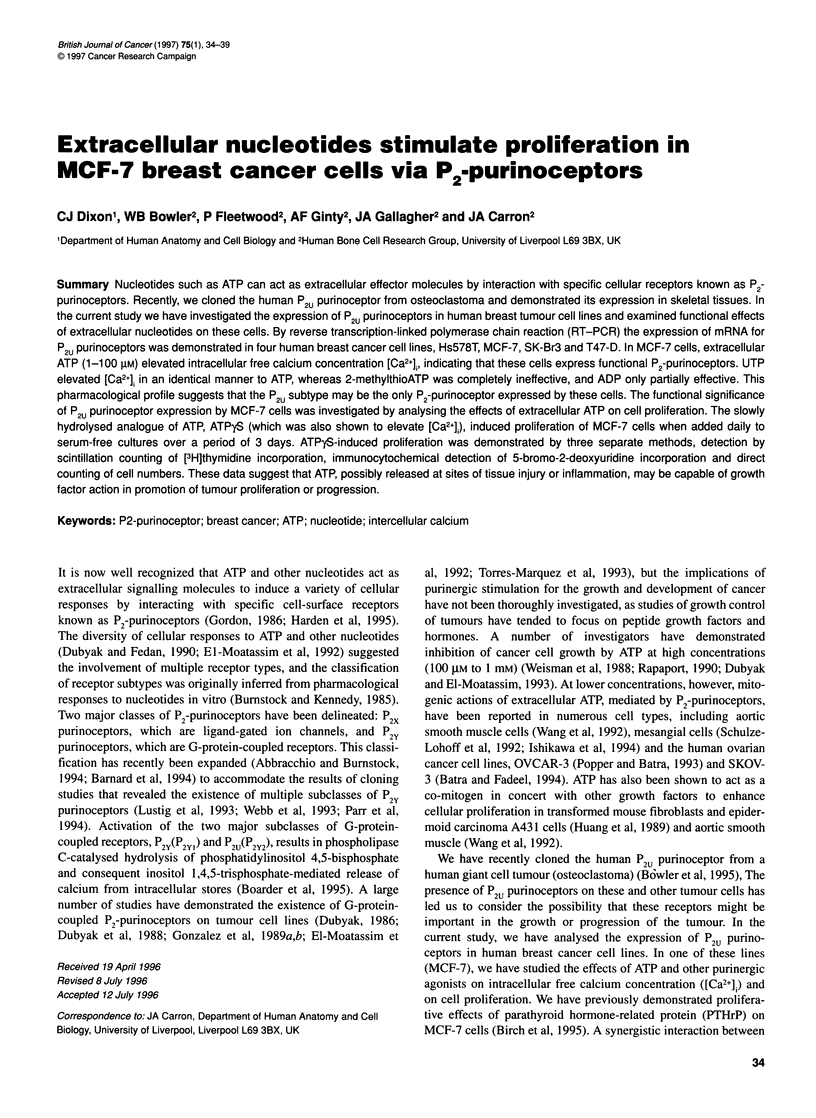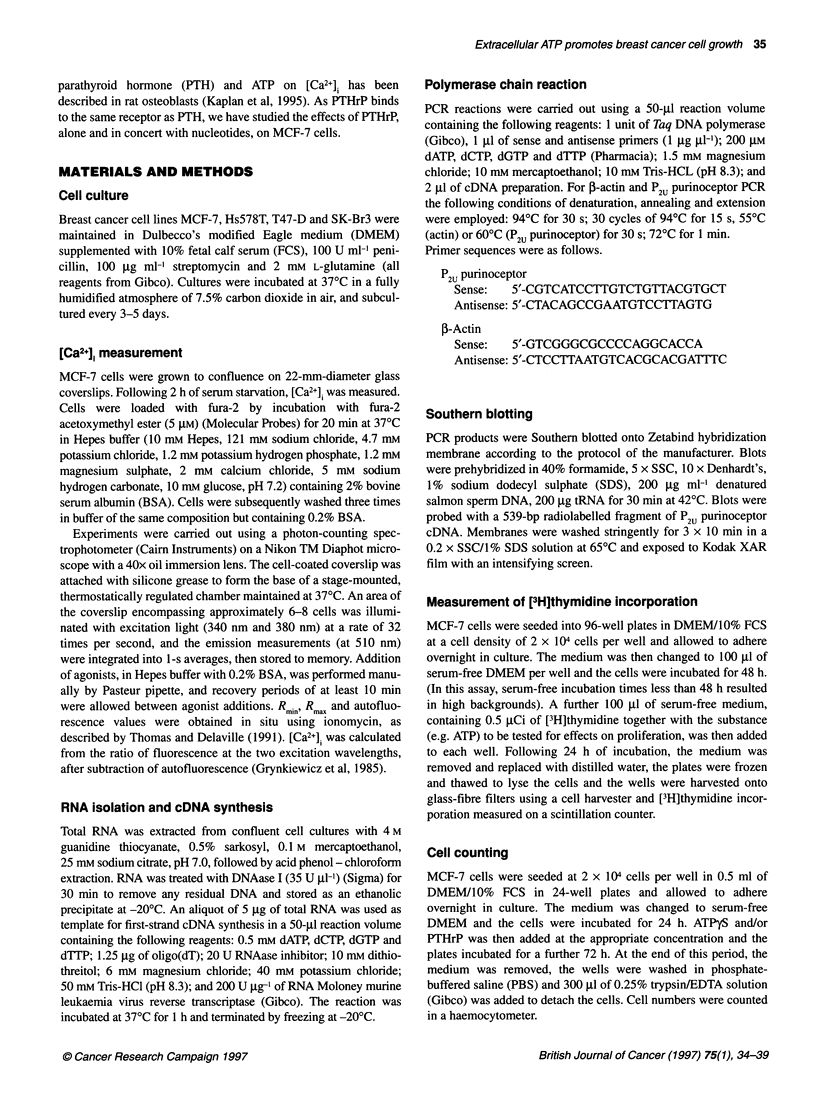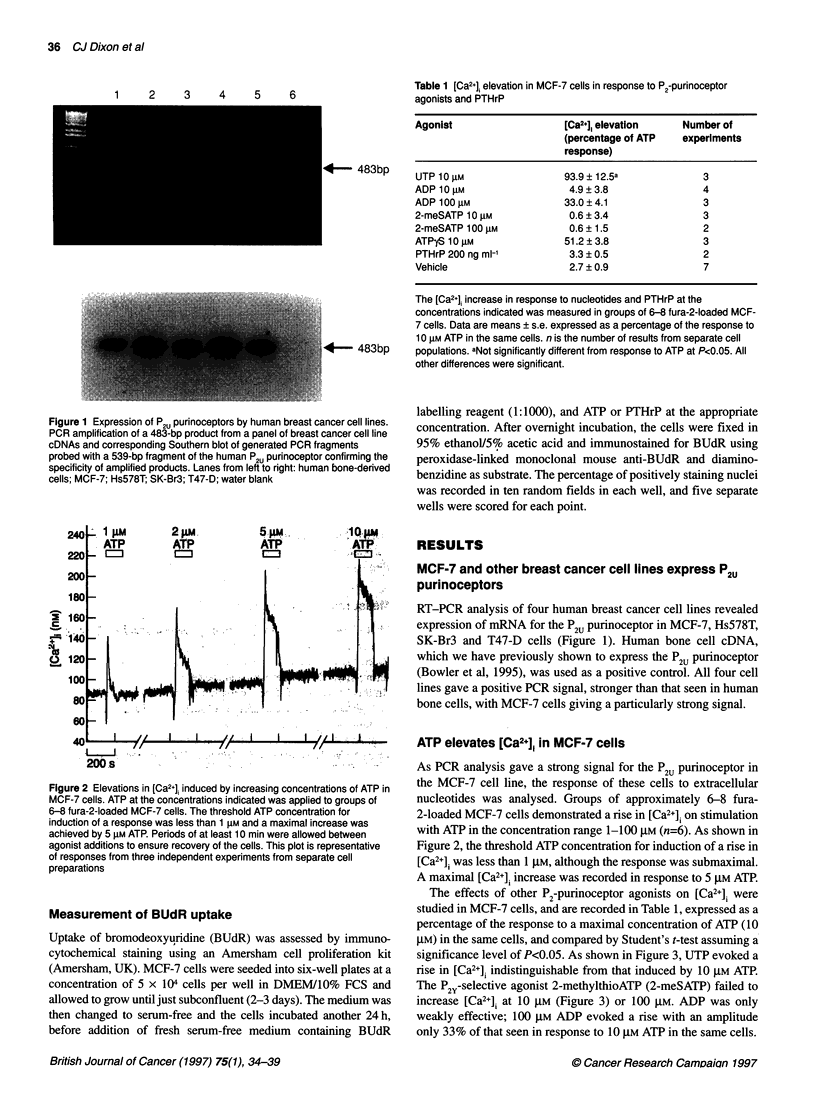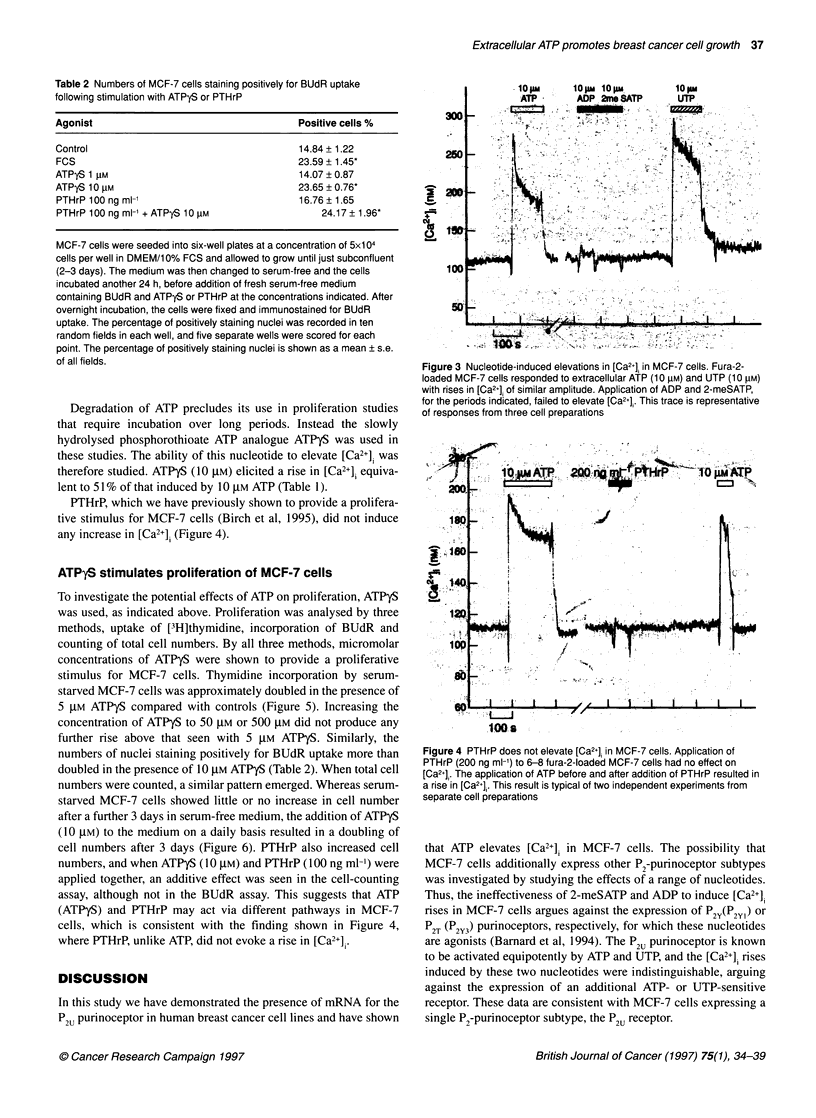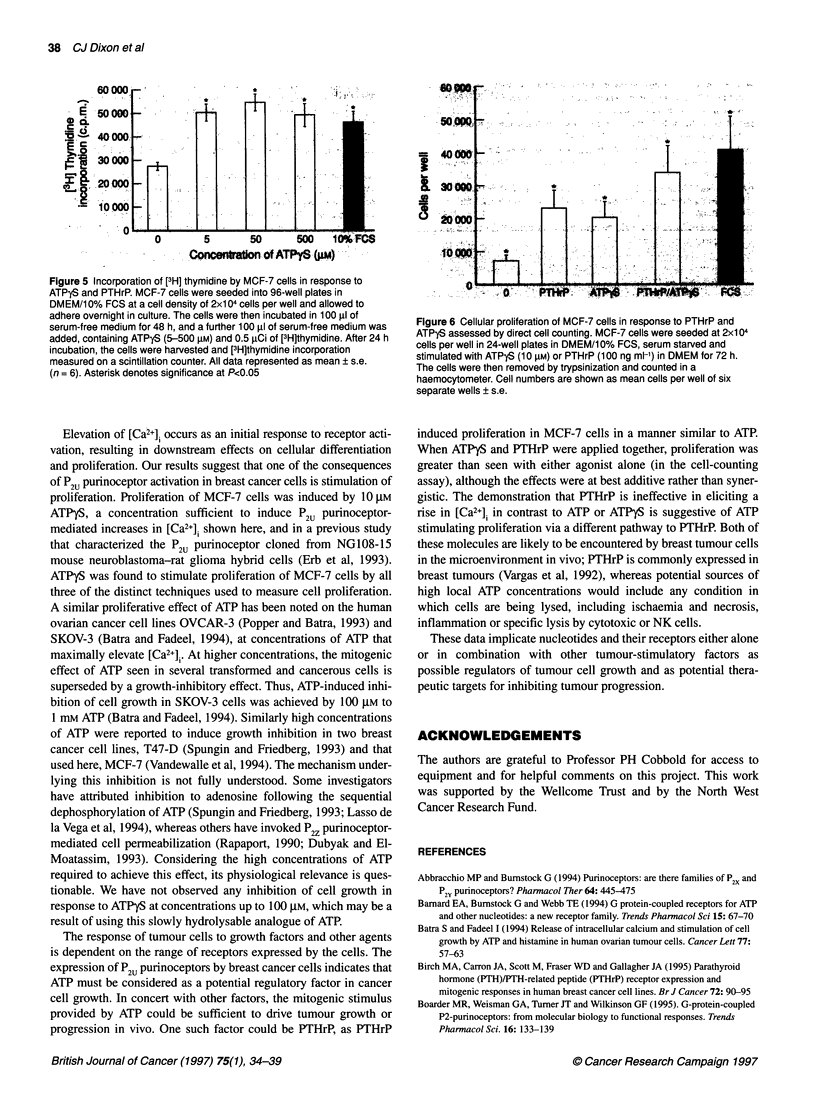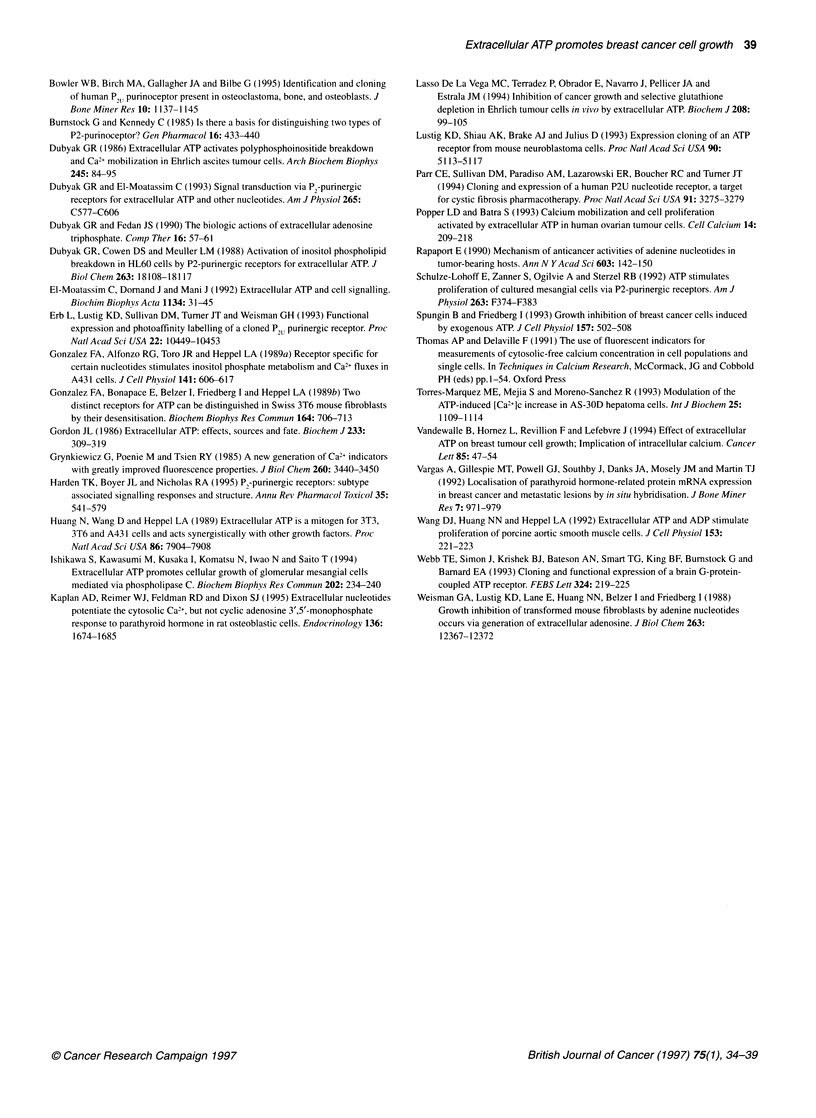# Extracellular nucleotides stimulate proliferation in MCF-7 breast cancer cells via P2-purinoceptors.

**DOI:** 10.1038/bjc.1997.6

**Published:** 1997

**Authors:** C. J. Dixon, W. B. Bowler, P. Fleetwood, A. F. Ginty, J. A. Gallagher, J. A. Carron

**Affiliations:** Department of Human Anatomy and Cell Biology, University of Liverpool, UK.

## Abstract

**Images:**


British Journal of Cancer (1997) 75(1), 34–39
© 1997 Cancer Research Campaign

### Extracellular nucleotides stimulate proliferation in MCF-7 breast cancer cells via $P_2$-purinoceptors

**CJ Dixon[1], WB Bowler[2], P Fleetwood[2], AF Ginty[2], JA Gallagher[2] and JA Carron[2]**

[1]Department of Human Anatomy and Cell Biology and [2]Human Bone Cell Research Group, University of Liverpool L69 3BX, UK

**Summary** Nucleotides such as ATP can act as extracellular effector molecules by interaction with specific cellular receptors known as $P_2$-purinoceptors. Recently, we cloned the human $P_{2U}$ purinoceptor from osteoclastoma and demonstrated its expression in skeletal tissues. In the current study we have investigated the expression of $P_{2U}$ purinoceptors in human breast tumour cell lines and examined functional effects of extracellular nucleotides on these cells. By reverse transcription-linked polymerase chain reaction (RT–PCR) the expression of mRNA for $P_{2U}$ purinoceptors was demonstrated in four human breast cancer cell lines, Hs578T, MCF-7, SK-Br3 and T47-D. In MCF-7 cells, extracellular ATP (1–100 μM) elevated intracellular free calcium concentration $[Ca^{2+}]_i$, indicating that these cells express functional $P_2$-purinoceptors. UTP elevated $[Ca^{2+}]_i$ in an identical manner to ATP, whereas 2-methylthioATP was completely ineffective, and ADP only partially effective. This pharmacological profile suggests that the $P_{2U}$ subtype may be the only $P_2$-purinoceptor expressed by these cells. The functional significance of $P_{2U}$ purinoceptor expression by MCF-7 cells was investigated by analysing the effects of extracellular ATP on cell proliferation. The slowly hydrolysed analogue of ATP, ATPγS (which was also shown to elevate $[Ca^{2+}]_i$), induced proliferation of MCF-7 cells when added daily to serum-free cultures over a period of 3 days. ATPγS-induced proliferation was demonstrated by three separate methods, detection by scintillation counting of [$^3$H]thymidine incorporation, immunocytochemical detection of 5-bromo-2-deoxyuridine incorporation and direct counting of cell numbers. These data suggest that ATP, possibly released at sites of tissue injury or inflammation, may be capable of growth factor action in promotion of tumour proliferation or progression.

**Keywords:** P2-purinoceptor; breast cancer; ATP; nucleotide; intercellular calcium

It is now well recognized that ATP and other nucleotides act as extracellular signalling molecules to induce a variety of cellular responses by interacting with specific cell-surface receptors known as $P_2$-purinoceptors (Gordon, 1986; Harden et al, 1995). The diversity of cellular responses to ATP and other nucleotides (Dubyak and Fedan, 1990; El-Moatassim et al, 1992) suggested the involvement of multiple receptor types, and the classification of receptor subtypes was originally inferred from pharmacological responses to nucleotides in vitro (Burnstock and Kennedy, 1985). Two major classes of $P_2$-purinoceptors have been delineated: $P_{2X}$ purinoceptors, which are ligand-gated ion channels, and $P_{2Y}$ purinoceptors, which are G-protein-coupled receptors. This classification has recently been expanded (Abbracchio and Burnstock, 1994; Barnard et al, 1994) to accommodate the results of cloning studies that revealed the existence of multiple subclasses of $P_{2Y}$ purinoceptors (Lustig et al, 1993; Webb et al, 1993; Parr et al, 1994). Activation of the two major subclasses of G-protein-coupled receptors, $P_{2Y}(P_{2Y1})$ and $P_{2U}(P_{2Y2})$, results in phospholipase C-catalysed hydrolysis of phosphatidylinositol 4,5-bisphosphate and consequent inositol 1,4,5-trisphosphate-mediated release of calcium from intracellular stores (Boarder et al, 1995). A large number of studies have demonstrated the existence of G-protein-coupled $P_2$-purinoceptors on tumour cell lines (Dubyak, 1986; Dubyak et al, 1988; Gonzalez et al, 1989*a*,*b*; El-Moatassim et al, 1992; Torres-Marquez et al, 1993), but the implications of purinergic stimulation for the growth and development of cancer have not been thoroughly investigated, as studies of growth control of tumours have tended to focus on peptide growth factors and hormones. A number of investigators have demonstrated inhibition of cancer cell growth by ATP at high concentrations (100 μM to 1 mM) (Weisman et al, 1988; Rapaport, 1990; Dubyak and El-Moatassim, 1993). At lower concentrations, however, mitogenic actions of extracellular ATP, mediated by $P_2$-purinoceptors, have been reported in numerous cell types, including aortic smooth muscle cells (Wang et al, 1992), mesangial cells (Schulze-Lohoff et al, 1992; Ishikawa et al, 1994) and the human ovarian cancer cell lines, OVCAR-3 (Popper and Batra, 1993) and SKOV-3 (Batra and Fadeel, 1994). ATP has also been shown to act as a co-mitogen in concert with other growth factors to enhance cellular proliferation in transformed mouse fibroblasts and epidermoid carcinoma A431 cells (Huang et al, 1989) and aortic smooth muscle (Wang et al, 1992).

*Received 19 April 1996*
*Revised 8 July 1996*
*Accepted 12 July 1996*

*Correspondence to:* JA Carron, Department of Human Anatomy and Cell Biology, University of Liverpool, Liverpool L69 3BX, UK

We have recently cloned the human $P_{2U}$ purinoceptor from a human giant cell tumour (osteoclastoma) (Bowler et al, 1995), The presence of $P_{2U}$ purinoceptors on these and other tumour cells has led us to consider the possibility that these receptors might be important in the growth or progression of the tumour. In the current study, we have analysed the expression of $P_{2U}$ purinoceptors in human breast cancer cell lines. In one of these lines (MCF-7), we have studied the effects of ATP and other purinergic agonists on intracellular free calcium concentration ($[Ca^{2+}]_i$) and on cell proliferation. We have previously demonstrated proliferative effects of parathyroid hormone-related protein (PTHrP) on MCF-7 cells (Birch et al, 1995). A synergistic interaction between parathyroid hormone (PTH) and ATP on $[Ca^{2+}]_i$ has been described in rat osteoblasts (Kaplan et al, 1995). As PTHrP binds to the same receptor as PTH, we have studied the effects of PTHrP, alone and in concert with nucleotides, on MCF-7 cells.

### MATERIALS AND METHODS

### Cell culture

Breast cancer cell lines MCF-7, Hs578T, T47-D and SK-Br3 were maintained in Dulbecco's modified Eagle medium (DMEM) supplemented with 10% fetal calf serum (FCS), 100 U $ml^{-1}$ penicillin, 100 μg $ml^{-1}$ streptomycin and 2 mM L-glutamine (all reagents from Gibco). Cultures were incubated at 37°C in a fully humidified atmosphere of 7.5% carbon dioxide in air, and subcultured every 3–5 days.

### $[Ca^{2+}]_i$ measurement

MCF-7 cells were grown to confluence on 22-mm-diameter glass coverslips. Following 2 h of serum starvation, $[Ca^{2+}]_i$ was measured. Cells were loaded with fura-2 by incubation with fura-2 acetoxymethyl ester (5 μM) (Molecular Probes) for 20 min at 37°C in Hepes buffer (10 mM Hepes, 121 mM sodium chloride, 4.7 mM potassium chloride, 1.2 mM potassium hydrogen phosphate, 1.2 mM magnesium sulphate, 2 mM calcium chloride, 5 mM sodium hydrogen carbonate, 10 mM glucose, pH 7.2) containing 2% bovine serum albumin (BSA). Cells were subsequently washed three times in buffer of the same composition but containing 0.2% BSA.

Experiments were carried out using a photon-counting spectrophotometer (Cairn Instruments) on a Nikon TM Diaphot microscope with a 40× oil immersion lens. The cell-coated coverslip was attached with silicone grease to form the base of a stage-mounted, thermostatically regulated chamber maintained at 37°C. An area of the coverslip encompassing approximately 6–8 cells was illuminated with excitation light (340 nm and 380 nm) at a rate of 32 times per second, and the emission measurements (at 510 nm) were integrated into 1-s averages, then stored to memory. Addition of agonists, in Hepes buffer with 0.2% BSA, was performed manually by Pasteur pipette, and recovery periods of at least 10 min were allowed between agonist additions. $R_{min}$, $R_{max}$ and autofluorescence values were obtained in situ using ionomycin, as described by Thomas and Delaville (1991). $[Ca^{2+}]_i$ was calculated from the ratio of fluorescence at the two excitation wavelengths, after subtraction of autofluorescence (Grynkiewicz et al, 1985).

### RNA isolation and cDNA synthesis

Total RNA was extracted from confluent cell cultures with 4 M guanidine thiocyanate, 0.5% sarkosyl, 0.1 M mercaptoethanol, 25 mM sodium citrate, pH 7.0, followed by acid phenol – chloroform extraction. RNA was treated with DNAase I (35 U $\mu l^{-1}$) (Sigma) for 30 min to remove any residual DNA and stored as an ethanolic precipitate at –20°C. An aliquot of 5 μg of total RNA was used as template for first-strand cDNA synthesis in a 50-μl reaction volume containing the following reagents: 0.5 mM dATP, dCTP, dGTP and dTTP; 1.25 μg of oligo(dT); 20 U RNAase inhibitor; 10 mM dithiothreitol; 6 mM magnesium chloride; 40 mM potassium chloride; 50 mM Tris-HCl (pH 8.3); and 200 U $\mu g^{-1}$ of RNA Moloney murine leukaemia virus reverse transcriptase (Gibco). The reaction was incubated at 37°C for 1 h and terminated by freezing at –20°C.

### Polymerase chain reaction

PCR reactions were carried out using a 50-μl reaction volume containing the following reagents: 1 unit of *Taq* DNA polymerase (Gibco), 1 μl of sense and antisense primers (1 μg $\mu l^{-1}$); 200 μM dATP, dCTP, dGTP and dTTP (Pharmacia); 1.5 mM magnesium chloride; 10 mM mercaptoethanol; 10 mM Tris-HCL (pH 8.3); and 2 μl of cDNA preparation. For β-actin and $P_{2U}$ purinoceptor PCR the following conditions of denaturation, annealing and extension were employed: 94°C for 30 s; 30 cycles of 94°C for 15 s, 55°C (actin) or 60°C ($P_{2U}$ purinoceptor) for 30 s; 72°C for 1 min. Primer sequences were as follows.

$P_{2U}$ purinoceptor
Sense: 5′-CGTCATCCTTGTCTGTTACGTGCT
Antisense: 5′-CTACAGCCGAATGTCCTTAGTG

β-Actin
Sense: 5′-GTCGGGCGCCCCAGGCACCA
Antisense: 5′-CTCCTTAATGTCACGCACGATTTC

### Southern blotting

PCR products were Southern blotted onto Zetabind hybridization membrane according to the protocol of the manufacturer. Blots were prehybridized in 40% formamide, 5 × SSC, 10 × Denhardt's, 1% sodium dodecyl sulphate (SDS), 200 μg $ml^{-1}$ denatured salmon sperm DNA, 200 μg tRNA for 30 min at 42°C. Blots were probed with a 539-bp radiolabelled fragment of $P_{2U}$ purinoceptor cDNA. Membranes were washed stringently for 3 × 10 min in a 0.2 × SSC/1% SDS solution at 65°C and exposed to Kodak XAR film with an intensifying screen.

### Measurement of [$^3$H]thymidine incorporation

MCF-7 cells were seeded into 96-well plates in DMEM/10% FCS at a cell density of $2 \times 10^4$ cells per well and allowed to adhere overnight in culture. The medium was then changed to 100 μl of serum-free DMEM per well and the cells were incubated for 48 h. (In this assay, serum-free incubation times less than 48 h resulted in high backgrounds). A further 100 μl of serum-free medium, containing 0.5 μCi of [$^3$H]thymidine together with the substance (e.g. ATP) to be tested for effects on proliferation, was then added to each well. Following 24 h of incubation, the medium was removed and replaced with distilled water, the plates were frozen and thawed to lyse the cells and the wells were harvested onto glass-fibre filters using a cell harvester and [$^3$H]thymidine incorporation measured on a scintillation counter.

### Cell counting

MCF-7 cells were seeded at $2 \times 10^4$ cells per well in 0.5 ml of DMEM/10% FCS in 24-well plates and allowed to adhere overnight in culture. The medium was changed to serum-free DMEM and the cells were incubated for 24 h. ATPγS and/or PTHrP was then added at the appropriate concentration and the plates incubated for a further 72 h. At the end of this period, the medium was removed, the wells were washed in phosphate-buffered saline (PBS) and 300 μl of 0.25% trypsin/EDTA solution (Gibco) was added to detach the cells. Cell numbers were counted in a haemocytometer.

© Cancer Research Campaign 1997

**Figure 1** Expression of $P_{2U}$ purinoceptors by human breast cancer cell lines. PCR amplification of a 483-bp product from a panel of breast cancer cell line cDNAs and corresponding Southern blot of generated PCR fragments probed with a 539-bp fragment of the human $P_{2U}$ purinoceptor confirming the specificity of amplified products. Lanes from left to right: human bone-derived cells; MCF-7; Hs578T; SK-Br3; T47-D; water blank

**Figure 2** Elevations in $[Ca^{2+}]_i$ induced by increasing concentrations of ATP in MCF-7 cells. ATP at the concentrations indicated was applied to groups of 6–8 fura-2-loaded MCF-7 cells. The threshold ATP concentration for induction of a response was less than 1 μM and a maximal increase was achieved by 5 μM ATP. Periods of at least 10 min were allowed between agonist additions to ensure recovery of the cells. This plot is representative of responses from three independent experiments from separate cell preparations

### Measurement of BUdR uptake

Uptake of bromodeoxyuridine (BUdR) was assessed by immunocytochemical staining using an Amersham cell proliferation kit (Amersham, UK). MCF-7 cells were seeded into six-well plates at a concentration of 5 × $10^4$ cells per well in DMEM/10% FCS and allowed to grow until just subconfluent (2–3 days). The medium was then changed to serum-free and the cells incubated another 24 h, before addition of fresh serum-free medium containing BUdR labelling reagent (1:1000), and ATP or PTHrP at the appropriate concentration. After overnight incubation, the cells were fixed in 95% ethanol/5% acetic acid and immunostained for BUdR using peroxidase-linked monoclonal mouse anti-BUdR and diaminobenzidine as substrate. The percentage of positively staining nuclei was recorded in ten random fields in each well, and five separate wells were scored for each point.

**Table 1** $[Ca^{2+}]_i$ elevation in MCF-7 cells in response to $P_2$-purinoceptor agonists and PTHrP

| Agonist | $[Ca^{2+}]_i$ elevation (percentage of ATP response) | Number of experiments |
|---|---|---|
| UTP 10 μM | 93.9 ± 12.5[a] | 3 |
| ADP 10 μM | 4.9 ± 3.8 | 4 |
| ADP 100 μM | 33.0 ± 4.1 | 3 |
| 2-meSATP 10 μM | 0.6 ± 3.4 | 3 |
| 2-meSATP 100 μM | 0.6 ± 1.5 | 2 |
| ATPγS 10 μM | 51.2 ± 3.8 | 3 |
| PTHrP 200 ng ml$^{-1}$ | 3.3 ± 0.5 | 2 |
| Vehicle | 2.7 ± 0.9 | 7 |

The $[Ca^{2+}]_i$ increase in response to nucleotides and PTHrP at the concentrations indicated was measured in groups of 6–8 fura-2-loaded MCF-7 cells. Data are means ± s.e. expressed as a percentage of the response to 10 μM ATP in the same cells. *n* is the number of results from separate cell populations. [a]Not significantly different from response to ATP at $P<0.05$. All other differences were significant.

### RESULTS

### MCF-7 and other breast cancer cell lines express $P_{2U}$ purinoceptors

RT–PCR analysis of four human breast cancer cell lines revealed expression of mRNA for the $P_{2U}$ purinoceptor in MCF-7, Hs578T, SK-Br3 and T47-D cells (Figure 1). Human bone cell cDNA, which we have previously shown to express the $P_{2U}$ purinoceptor (Bowler et al, 1995), was used as a positive control. All four cell lines gave a positive PCR signal, stronger than that seen in human bone cells, with MCF-7 cells giving a particularly strong signal.

### ATP elevates $[Ca^{2+}]_i$ in MCF-7 cells

As PCR analysis gave a strong signal for the $P_{2U}$ purinoceptor in the MCF-7 cell line, the response of these cells to extracellular nucleotides was analysed. Groups of approximately 6–8 fura-2-loaded MCF-7 cells demonstrated a rise in $[Ca^{2+}]_i$ on stimulation with ATP in the concentration range 1–100 μM ($n$=6). As shown in Figure 2, the threshold ATP concentration for induction of a rise in $[Ca^{2+}]_i$ was less than 1 μM, although the response was submaximal. A maximal $[Ca^{2+}]_i$ increase was recorded in response to 5 μM ATP.

The effects of other $P_2$-purinoceptor agonists on $[Ca^{2+}]_i$ were studied in MCF-7 cells, and are recorded in Table 1, expressed as a percentage of the response to a maximal concentration of ATP (10 μM) in the same cells, and compared by Student's *t*-test assuming a significance level of $P<0.05$. As shown in Figure 3, UTP evoked a rise in $[Ca^{2+}]_i$ indistinguishable from that induced by 10 μM ATP. The $P_{2Y}$-selective agonist 2-methylthioATP (2-meSATP) failed to increase $[Ca^{2+}]_i$ at 10 μM (Figure 3) or 100 μM. ADP was only weakly effective; 100 μM ADP evoked a rise with an amplitude only 33% of that seen in response to 10 μM ATP in the same cells.

© Cancer Research Campaign 1997

**Table 2** Numbers of MCF-7 cells staining positively for BUdR uptake following stimulation with ATPγS or PTHrP

| Agonist | Positive cells % |
|---|---|
| Control | 14.84 ± 1.22 |
| FCS | 23.59 ± 1.45* |
| ATPγS 1 μM | 14.07 ± 0.87 |
| ATPγS 10 μM | 23.65 ± 0.76* |
| PTHrP 100 ng ml$^{-1}$ | 16.76 ± 1.65 |
| PTHrP 100 ng ml$^{-1}$ + ATPγS 10 μM | 24.17 ± 1.96* |

MCF-7 cells were seeded into six-well plates at a concentration of 5×10$^4$ cells per well in DMEM/10% FCS and allowed to grow until just subconfluent (2–3 days). The medium was then changed to serum-free and the cells incubated another 24 h, before addition of fresh serum-free medium containing BUdR and ATPγS or PTHrP at the concentrations indicated. After overnight incubation, the cells were fixed and immunostained for BUdR uptake. The percentage of positively staining nuclei was recorded in ten random fields in each well, and five separate wells were scored for each point. The percentage of positively staining nuclei is shown as a mean ± s.e. of all fields.

Degradation of ATP precludes its use in proliferation studies that require incubation over long periods. Instead the slowly hydrolysed phosphorothioate ATP analogue ATPγS was used in these studies. The ability of this nucleotide to elevate $[Ca^{2+}]_i$ was therefore studied. ATPγS (10 μM) elicited a rise in $[Ca^{2+}]_i$ equivalent to 51% of that induced by 10 μM ATP (Table 1).

PTHrP, which we have previously shown to provide a proliferative stimulus for MCF-7 cells (Birch et al, 1995), did not induce any increase in $[Ca^{2+}]_i$ (Figure 4).

### ATPγS stimulates proliferation of MCF-7 cells

To investigate the potential effects of ATP on proliferation, ATPγS was used, as indicated above. Proliferation was analysed by three methods, uptake of [$^3$H]thymidine, incorporation of BUdR and counting of total cell numbers. By all three methods, micromolar concentrations of ATPγS were shown to provide a proliferative stimulus for MCF-7 cells. Thymidine incorporation by serum-starved MCF-7 cells was approximately doubled in the presence of 5 μM ATPγS compared with controls (Figure 5). Increasing the concentration of ATPγS to 50 μM or 500 μM did not produce any further rise above that seen with 5 μM ATPγS. Similarly, the numbers of nuclei staining positively for BUdR uptake more than doubled in the presence of 10 μM ATPγS (Table 2). When total cell numbers were counted, a similar pattern emerged. Whereas serum-starved MCF-7 cells showed little or no increase in cell number after a further 3 days in serum-free medium, the addition of ATPγS (10 μM) to the medium on a daily basis resulted in a doubling of cell numbers after 3 days (Figure 6). PTHrP also increased cell numbers, and when ATPγS (10 μM) and PTHrP (100 ng ml$^{-1}$) were applied together, an additive effect was seen in the cell-counting assay, although not in the BUdR assay. This suggests that ATP (ATPγS) and PTHrP may act via different pathways in MCF-7 cells, which is consistent with the finding shown in Figure 4, where PTHrP, unlike ATP, did not evoke a rise in $[Ca^{2+}]_i$.

### DISCUSSION

In this study we have demonstrated the presence of mRNA for the $P_{2U}$ purinoceptor in human breast cancer cell lines and have shown

**Figure 3** Nucleotide-induced elevations in $[Ca^{2+}]_i$ in MCF-7 cells. Fura-2-loaded MCF-7 cells responded to extracellular ATP (10 μM) and UTP (10 μM) with rises in $[Ca^{2+}]_i$ of similar amplitude. Application of ADP and 2-meSATP, for the periods indicated, failed to elevate $[Ca^{2+}]_i$. This trace is representative of responses from three cell preparations

**Figure 4** PTHrP does not elevate $[Ca^{2+}]_i$ in MCF-7 cells. Application of PTHrP (200 ng ml$^{-1}$) to 6–8 fura-2-loaded MCF-7 cells had no effect on $[Ca^{2+}]_i$. The application of ATP before and after addition of PTHrP resulted in a rise in $[Ca^{2+}]_i$. This result is typical of two independent experiments from separate cell preparations that ATP elevates $[Ca^{2+}]_i$ in MCF-7 cells. The possibility that MCF-7 cells additionally express other $P_2$-purinoceptor subtypes was investigated by studying the effects of a range of nucleotides. Thus, the ineffectiveness of 2-meSATP and ADP to induce $[Ca^{2+}]_i$ rises in MCF-7 cells argues against the expression of $P_{2Y}(P_{2Y1})$ or $P_{2T}$ ($P_{2Y3}$) purinoceptors, respectively, for which these nucleotides are agonists (Barnard et al, 1994). The $P_{2U}$ purinoceptor is known to be activated equipotently by ATP and UTP, and the $[Ca^{2+}]_i$ rises induced by these two nucleotides were indistinguishable, arguing against the expression of an additional ATP- or UTP-sensitive receptor. These data are consistent with MCF-7 cells expressing a single $P_2$-purinoceptor subtype, the $P_{2U}$ receptor.

© Cancer Research Campaign 1997

**Figure 5** Incorporation of [$^3$H] thymidine by MCF-7 cells in response to ATPγS and PTHrP. MCF-7 cells were seeded into 96-well plates in DMEM/10% FCS at a cell density of $2 \times 10^4$ cells per well and allowed to adhere overnight in culture. The cells were then incubated in 100 μl of serum-free medium for 48 h, and a further 100 μl of serum-free medium was added, containing ATPγS (5–500 μM) and 0.5 μCi of [$^3$H]thymidine. After 24 h incubation, the cells were harvested and [$^3$H]thymidine incorporation measured on a scintillation counter. All data represented as mean ± s.e. ($n$ = 6). Asterisk denotes significance at $P$<0.05

**Figure 6** Cellular proliferation of MCF-7 cells in response to PTHrP and ATPγS assessed by direct cell counting. MCF-7 cells were seeded at $2 \times 10^4$ cells per well in 24-well plates in DMEM/10% FCS, serum starved and stimulated with ATPγS (10 μM) or PTHrP (100 ng $ml^{-1}$) in DMEM for 72 h. The cells were then removed by trypsinization and counted in a haemocytometer. Cell numbers are shown as mean cells per well of six separate wells ± s.e.

Elevation of $[Ca^{2+}]_i$ occurs as an initial response to receptor activation, resulting in downstream effects on cellular differentiation and proliferation. Our results suggest that one of the consequences of $P_{2U}$ purinoceptor activation in breast cancer cells is stimulation of proliferation. Proliferation of MCF-7 cells was induced by 10 μM ATPγS, a concentration sufficient to induce $P_{2U}$ purinoceptor-mediated increases in $[Ca^{2+}]_i$ shown here, and in a previous study that characterized the $P_{2U}$ purinoceptor cloned from NG108-15 mouse neuroblastoma–rat glioma hybrid cells (Erb et al, 1993). ATPγS was found to stimulate proliferation of MCF-7 cells by all three of the distinct techniques used to measure cell proliferation. A similar proliferative effect of ATP has been noted on the human ovarian cancer cell lines OVCAR-3 (Popper and Batra, 1993) and SKOV-3 (Batra and Fadeel, 1994), at concentrations of ATP that maximally elevate $[Ca^{2+}]_i$. At higher concentrations, the mitogenic effect of ATP seen in several transformed and cancerous cells is superseded by a growth-inhibitory effect. Thus, ATP-induced inhibition of cell growth in SKOV-3 cells was achieved by 100 μM to 1 mM ATP (Batra and Fadeel, 1994). Similarly high concentrations of ATP were reported to induce growth inhibition in two breast cancer cell lines, T47-D (Spungin and Friedberg, 1993) and that used here, MCF-7 (Vandewalle et al, 1994). The mechanism underlying this inhibition is not fully understood. Some investigators have attributed inhibition to adenosine following the sequential dephosphorylation of ATP (Spungin and Friedberg, 1993; Lasso de la Vega et al, 1994), whereas others have invoked $P_{2Z}$ purinoceptor-mediated cell permeabilization (Rapaport, 1990; Dubyak and El-Moatassim, 1993). Considering the high concentrations of ATP required to achieve this effect, its physiological relevance is questionable. We have not observed any inhibition of cell growth in response to ATPγS at concentrations up to 100 μM, which may be a result of using this slowly hydrolysable analogue of ATP.

The response of tumour cells to growth factors and other agents is dependent on the range of receptors expressed by the cells. The expression of $P_{2U}$ purinoceptors by breast cancer cells indicates that ATP must be considered as a potential regulatory factor in cancer cell growth. In concert with other factors, the mitogenic stimulus provided by ATP could be sufficient to drive tumour growth or progression in vivo. One such factor could be PTHrP, as PTHrP induced proliferation in MCF-7 cells in a manner similar to ATP. When ATPγS and PTHrP were applied together, proliferation was greater than seen with either agonist alone (in the cell-counting assay), although the effects were at best additive rather than synergistic. The demonstration that PTHrP is ineffective in eliciting a rise in $[Ca^{2+}]_i$ in contrast to ATP or ATPγS is suggestive of ATP stimulating proliferation via a different pathway to PTHrP. Both of these molecules are likely to be encountered by breast tumour cells in the microenvironment in vivo; PTHrP is commonly expressed in breast tumours (Vargas et al, 1992), whereas potential sources of high local ATP concentrations would include any condition in which cells are being lysed, including ischaemia and necrosis, inflammation or specific lysis by cytotoxic or NK cells.

These data implicate nucleotides and their receptors either alone or in combination with other tumour-stimulatory factors as possible regulators of tumour cell growth and as potential therapeutic targets for inhibiting tumour progression.

### ACKNOWLEDGEMENTS

The authors are grateful to Professor PH Cobbold for access to equipment and for helpful comments on this project. This work was supported by the Wellcome Trust and by the North West Cancer Research Fund.

### REFERENCES

Abbracchio MP and Burnstock G (1994) Purinoceptors: are there families of $P_{2X}$ and $P_{2Y}$ purinoceptors? *Pharmacol Ther* **64:** 445–475

Barnard EA, Burnstock G and Webb TE (1994) G protein-coupled receptors for ATP and other nucleotides: a new receptor family. *Trends Pharmacol Sci* **15:** 67–70

Batra S and Fadeel I (1994) Release of intracellular calcium and stimulation of cell growth by ATP and histamine in human ovarian tumour cells. *Cancer Lett* **77:** 57–63

Birch MA, Carron JA, Scott M, Fraser WD and Gallagher JA (1995) Parathyroid hormone (PTH)/PTH-related peptide (PTHrP) receptor expression and mitogenic responses in human breast cancer cell lines. *Br J Cancer* **72:** 90–95

Boarder MR, Weisman GA, Turner JT and Wilkinson GF (1995). G-protein-coupled P2-purinoceptors: from molecular biology to functional responses. *Trends Pharmacol Sci.* **16:** 133–139

© Cancer Research Campaign 1997

Bowler WB, Birch MA, Gallagher JA and Bilbe G (1995) Identification and cloning of human $P_{2U}$ purinoceptor present in osteoclastoma, bone, and osteoblasts. *J Bone Miner Res* **10:** 1137–1145

Burnstock G and Kennedy C (1985) Is there a basis for distinguishing two types of P2-purinoceptor? *Gen Pharmacol* **16:** 433–440

Dubyak GR (1986) Extracellular ATP activates polyphosphoinositide breakdown and $Ca^{2+}$ mobilization in Ehrlich ascites tumour cells. *Arch Biochem Biophys* **245:** 84–95

Dubyak GR and El-Moatassim C (1993) Signal transduction via $P_2$-purinergic receptors for extracellular ATP and other nucleotides. *Am J Physiol* **265:** C577–C606

Dubyak GR and Fedan JS (1990) The biologic actions of extracellular adenosine triphosphate. *Comp Ther* **16:** 57–61

Dubyak GR, Cowen DS and Meuller LM (1988) Activation of inositol phospholipid breakdown in HL60 cells by P2-purinergic receptors for extracellular ATP. *J Biol Chem* **263:** 18108–18117

El-Moatassim C, Dornand J and Mani J (1992) Extracellular ATP and cell signalling. *Biochim Biophys Acta* **1134:** 31–45

Erb L, Lustig KD, Sullivan DM, Turner JT and Weisman GH (1993) Functional expression and photoaffinity labelling of a cloned $P_{2U}$ purinergic receptor. *Proc Natl Acad Sci USA* **22:** 10449–10453

Gonzalez FA, Alfonzo RG, Toro JR and Heppel LA (1989*a*) Receptor specific for certain nucleotides stimulates inositol phosphate metabolism and $Ca^{2+}$ fluxes in A431 cells. *J Cell Physiol* **141:** 606–617

Gonzalez FA, Bonapace E, Belzer I, Friedberg I and Heppel LA (1989*b*) Two distinct receptors for ATP can be distinguished in Swiss 3T6 mouse fibroblasts by their desensitisation. *Biochem Biophys Res Commun* **164:** 706–713

Gordon JL (1986) Extracellular ATP: effects, sources and fate. *Biochem J* **233:** 309–319

Grynkiewicz G, Poenie M and Tsien RY (1985) A new generation of $Ca^{2+}$ indicators with greatly improved fluorescence properties. *J Biol Chem* **260:** 3440–3450

Harden TK, Boyer JL and Nicholas RA (1995) $P_2$-purinergic receptors: subtype associated signalling responses and structure. *Annu Rev Pharmacol Toxicol* **35:** 541–579

Huang N, Wang D and Heppel LA (1989) Extracellular ATP is a mitogen for 3T3, 3T6 and A431 cells and acts synergistically with other growth factors. *Proc Natl Acad Sci USA* **86:** 7904–7908

Ishikawa S, Kawasumi M, Kusaka I, Komatsu N, Iwao N and Saito T (1994) Extracellular ATP promotes cellular growth of glomerular mesangial cells mediated via phospholipase C. *Biochem Biophys Res Commun* **202:** 234–240

Kaplan AD, Reimer WJ, Feldman RD and Dixon SJ (1995) Extracellular nucleotides potentiate the cytosolic $Ca^{2+}$, but not cyclic adenosine 3′,5′-monophosphate response to parathyroid hormone in rat osteoblastic cells. *Endocrinology* **136:** 1674–1685

Lasso De La Vega MC, Terradez P, Obrador E, Navarro J, Pellicer JA and Estrala JM (1994) Inhibition of cancer growth and selective glutathione depletion in Ehrlich tumour cells *in vivo* by extracellular ATP. *Biochem J* **208:** 99–105

Lustig KD, Shiau AK, Brake AJ and Julius D (1993) Expression cloning of an ATP receptor from mouse neuroblastoma cells. *Proc Natl Acad Sci USA* **90:** 5113–5117

Parr CE, Sullivan DM, Paradiso AM, Lazarowski ER, Boucher RC and Turner JT (1994) Cloning and expression of a human P2U nucleotide receptor, a target for cystic fibrosis pharmacotherapy. *Proc Natl Acad Sci USA* **91:** 3275–3279

Popper LD and Batra S (1993) Calcium mobilization and cell proliferation activated by extracellular ATP in human ovarian tumour cells. *Cell Calcium* **14:** 209–218

Rapaport E (1990) Mechanism of anticancer activities of adenine nucleotides in tumor-bearing hosts. *Ann N Y Acad Sci* **603:** 142–150

Schulze-Lohoff E, Zanner S, Ogilvie A and Sterzel RB (1992) ATP stimulates proliferation of cultured mesangial cells via P2-purinergic receptors. *Am J Physiol* **263:** F374–F383

Spungin B and Friedberg I (1993) Growth inhibition of breast cancer cells induced by exogenous ATP. *J Cell Physiol* **157:** 502–508

Thomas AP and Delaville F (1991) The use of fluorescent indicators for measurements of cytosolic-free calcium concentration in cell populations and single cells. In *Techniques in Calcium Research*, McCormack, JG and Cobbold PH (eds) pp.1–54. Oxford Press

Torres-Marquez ME, Mejia S and Moreno-Sanchez R (1993) Modulation of the ATP-induced $[Ca^{2+}]c$ increase in AS-30D hepatoma cells. *Int J Biochem* **25:** 1109–1114

Vandewalle B, Hornez L, Revillion F and Lefebvre J (1994) Effect of extracellular ATP on breast tumour cell growth; Implication of intracellular calcium. *Cancer Lett* **85:** 47–54

Vargas A, Gillespie MT, Powell GJ, Southby J, Danks JA, Mosely JM and Martin TJ (1992) Localisation of parathyroid hormone-related protein mRNA expression in breast cancer and metastatic lesions by *in situ* hybridisation. *J Bone Miner Res* **7:** 971–979

Wang DJ, Huang NN and Heppel LA (1992) Extracellular ATP and ADP stimulate proliferation of porcine aortic smooth muscle cells. *J Cell Physiol* **153:** 221–223

Webb TE, Simon J, Krishek BJ, Bateson AN, Smart TG, King BF, Burnstock G and Barnard EA (1993) Cloning and functional expression of a brain G-protein-coupled ATP receptor. *FEBS Lett* **324:** 219–225

Weisman GA, Lustig KD, Lane E, Huang NN, Belzer I and Friedberg I (1988) Growth inhibition of transformed mouse fibroblasts by adenine nucleotides occurs via generation of extracellular adenosine. *J Biol Chem* **263:** 12367–12372

*© Cancer Research Campaign 1997*